# Neonatal mortality and associated factors among newborns in Mogadishu, Somalia: a multicenter hospital-based cross-sectional study

**DOI:** 10.1186/s12889-024-19149-7

**Published:** 2024-06-19

**Authors:** Ikran Abdulkadir Ali, Pamornsri Inchon, Sirinan Suwannaporn, Jullapong Achalapong

**Affiliations:** 1 Department of Public Health, School of Health Science, Mae Fah Luang University, Chiang Rai Province, Thailand; 2 Department of Neonatal Intensive Care Unit, Yardimeli Hospital, Mogadishu, Somalia; 3 School of Medicine, Mae Fah Luang University, Chiang Rai Province, Thailand

**Keywords:** Associated factors, Neonatal mortality, Newborns, Prevalence, Mogadishu, Somalia

## Abstract

**Introduction:**

Neonatal mortality is a significant public health problem in Sub-Saharan Africa, particularly in Somalia, where limited data exists about this. Mogadishu, the densely populated capital, faces a high rate of neonatal mortality, but this has not been widely studied on a national level. Healthcare providers and policymakers are working to reduce newborn deaths, but a comprehensive understanding of the contributing factors is crucial for effective strategies. Therefore, this study aims to determine the magnitude of neonatal death and identify factors associated with it in Mogadishu, Somalia.

**Method:**

A multicenter hospital-based cross-sectional study was conducted to collect data from participants at 5 purposively selected hospitals in Mogadishu, Somalia. A well-structured, reliable, self-developed, validated questionnaire containing socio-demographic, maternal, and neonatal characteristics was used as a research tool. Descriptive statistics were used for categorical and continuous variables presented. Chi-square and logistic regression were used to identify factors associated with neonatal mortality at a significant level of α = 0.05.

**Results:**

A total of 513 participants were recruited for the study. The prevalence of neonatal mortality was 26.5% [95%CI = 22.6–30.2]. In a multivariable model, 9 variables were found: female newborns (AOR = 1.98, 95%CI = 1.22–3.19), those their mothers who did not attend ANC visits (AOR = 2.59, 95%CI = 1.05–6.45), those their mothers who did not take tetanus toxoid vaccination (AOR = 1.82, 95%CI = 1.01–3.28), those their mothers who delivered in instrumental assistant mode (AOR = 3.01, 95%CI = 1.38–6.56), those who had neonatal sepsis (AOR = 2.24, (95%CI = 1.26–3.98), neonatal tetanus (AOR = 16.03, 95%CI = 3.69–69.49), and pneumonia (AOR = 4.06, 95%CI = 1.60–10.31) diseases during hospitalization, premature (AOR = 1.99, 95%CI = 1.00–3.94) and postmature (AOR = 4.82, 95%CI = 1.64–14.16) neonates, those with a birth weight of less than 2500 gr (AOR = 4.82, 95%CI = 2.34–9.95), those who needed resuscitation after delivery (AOR = 2.78, 95%CI = 1.51–5.13), and those who did not initiate early breastfeeding (AOR = 2.28, 95%CI = 1.12–4.66), were significantly associated with neonatal mortality compared to their counterparts.

**Conclusion:**

In this study, neonatal mortality was high prevalence. Therefore, the intervention efforts should focus on strategies to reduce maternal and neonatal factors related to neonatal mortality. Healthcare workers and health institutions should provide appropriate antenatal, postnatal, and newborn care.

## Background

Neonatal mortality (NM) refers to the occurrence of a newborn’s death within the initial 28 days of life [1]. These initial 28 days are a crucial phase in a baby’s life, making them more susceptible to opportunistic infections and other health challenges [2, 3]. One-third of all neonatal deaths occur within the first day of life, 75.0% transpiring within the initial week after birth, underscoring the critical importance of the first 24 h [4]. In the year 2021, on a global scale, 2.3 million children lost their lives in the first month after birth, translating to approximately 6,400 neonatal fatalities each day [5]. Surprisingly, 99.0% of these newborn deaths occurred in low and middle-income countries, with Southern Asia and Sub-Saharan Africa having the highest burden of neonatal mortalities [6–8]. Globally, over 30 million neonatal deaths are predicted to occur between 2017 and 2030, and almost half of these deaths are expected to occur within the Sub-Saharan African region [9].

Neonatal mortality (NM) remains a public health concern in underdeveloped countries [10, 11]. Despite progress in reducing NM over the last three decades, efforts to improve progress are still required to meet the 2030 SDG target [12]. Neonatal deaths comprise more than half of all under-five deaths worldwide [13]. Consequently, prematurity, complications during labor, and neonatal infections contribute to 75% of neonatal mortality [14, 15]. Although there is a global increase in newborn mortality, the burden is greatest in West and Central Africa, where the probability of a baby dying in the first 28 days of life is over ten times that of high-income countries [16]. Furthermore, resource-limited countries are falling short of meeting the World Health Organization (WHO) standards, resulting in an increased toll of newborn and maternal mortality due to inadequate healthcare systems [17]. The prevalence of neonatal mortality in Sub-Saharan Africa and Southern Asia accounts for 79% of all newborn deaths in low-middle-income countries [8]. The rates of neonatal mortality vary significantly across different countries, with Ghana reporting a rate of 20.2% [18], India at 26.6% [19], Congo at 47% [20], Ethiopia at 35.5% [21], and Pakistan at 49% [22]. Furthermore, the WHO highlighted the high neonatal mortality rate in South Africa’s Lesotho province in 2019. This alarming rate was attributed to a shortage of essential medical supplies and harsh environmental conditions [23].

Neonatal mortality is primarily influenced by several critical factors, including a deficiency in high-quality intrapartum care, birth asphyxia, premature birth, infections, and birth abnormalities [4]. These factors account for most neonatal deaths within the first week of life [4]. Furthermore, early gestational age, parents with lower levels of education, inadequate primary antenatal care, low birth weight, the need for resuscitation, hypothermia, respiratory distress syndrome, an Apgar score below 7, and maternal age under 20 years were also reported [24–32].

In Somalia, there is limited data about neonatal deaths and their associated factors, particularly in Mogadishu, the capital and most densely populated city. Maternal and newborn healthcare coverage is inadequate. While 38.4% of newborn deaths were reported decades ago, resulting in insufficient healthcare and limited socio-economic development [33, 34]. Furthermore, the utilization of prenatal care services falls short, with only 26.0% of expectant mothers receiving at least one visit during their last pregnancy, which might lead to adverse pregnancy outcomes with higher neonatal mortality [35]. A previous Somalian study showed a high prevalence of premature births with a high incidence of maternal complications that can enhance the risk of neonatal mortality [36]. The vulnerability of Somali newborns and mothers is exacerbated by the inadequacy and inaccessibility of medical services [37].

In addition, clinics in Mogadishu have reported a high incidence of neonatal deaths, which has not been scientifically documented on a national scale. Despite these challenges, healthcare providers and policymakers in Somalia have directed their efforts to establish an effective mechanism to reduce newborn deaths. However, these efforts may not produce meaningful results without a comprehensive understanding of the factors contributing to neonatal mortality. Thus, it is imperative to gather up-to-date information to bolster prevention strategies, provide crucial support to policymakers, and implement the necessary medical and public health interventions at both the national and international levels. However, our study aims to determine the magnitude of neonatal death and identify factors associated with it in Mogadishu, Somalia.

## Methods and materials

### Study design and setting

A cross-sectional multicenter study was conducted in Mogadishu, Somalia, from January to April 2023. Mogadishu, the capital and most densely populated city contains 17 districts and has a population exceeding 3 million individuals. Although the city has over 30 hospitals, few are equipped with neonatal intensive care units (NICUs). This research purposively selected five hospitals based on the availability of NICUs, their status as referral hospitals, their number of admission cases, their substantial patient influx, specialized departments or units, and their capacity to offer a wide range of services for NICU and newborn care. These were Banadir Hospital, Yardimeli Hospital, Somali Sudanese Specialist Hospital, SOS Hospital, and Furqan Hospital.

### Study population and eligibility criteria

This study enrolled all newborns aged 28 days or younger admitted to the Neonatal Intensive Care Unit (NICU) and newborn care departments of selected hospitals and their mothers during the study period after becoming eligible for the study. Exclusions were made for those with incomplete admission records, self-discharged patients from the NICU and newborn care units, and those initially admitted, as well as newborn cases where mothers were unaware of their previous pregnancy and childbirth histories.

### Sample size and sampling technique

This study’s sample size was calculated using standardized cross-sectional Cochran’s formula: n= $\frac{{{\varvec{z}}}^{2}\mathbf{p}\mathbf{q}}{{{\varvec{e}}}^{2}}$ [38]. n = desired sample size, z = standard normal distribution at the desired confidence level (1.96), q = 1-p, e = desired level of precision; e = 0.05, and p = prevalence of neonatal mortality (35.5%) from a previous study in Ethiopia [21]. Therefore, our study required a total sample size of 387 respondents, including 10% non-response. However, this study initially recruited 524 participants, and then 11 respondents were excluded due to incomplete case record files, refusal to participate in interviews, and duplicate admissions. Therefore, the final sample in this study is 513 participants. Simple random sampling techniques were used to select the study participants. The Probability Proportional to Size (PPS) technique [39] was used to distribute samples in a manner that accurately reflects the proportional allocation in each hospital. This resulted in the selection of 176 cases from Banadir Hospital, 136 cases from Yardimeli Hospital, 71 cases from Somali-Sudanese Hospital, 72 cases from Furqan Hospital, and 58 cases from SOS Hospital.

### Research instrument development and data quality

A well-structured, self-developed, reliable questionnaire was developed from an extensive literature review and then discussed by field experts [27, 28, 32, 40]. The questionnaire was initially developed in English and then verbally translated during the data collection. Content validity was assessed using the Item Objective Congruence (IOC) method by three external experts [41]. Questions that received a score below 0.5 were removed from the questionnaire, those scored between 0.5 and 0.7 were revised based on expert feedback, and questions scoring above 0.7 were incorporated into the questionnaire without alterations. Furthermore, a pilot study containing 30 responses from five selected hospitals with similar characteristics was conducted to enhance the questionnaire’s reliability and the respondents’ understanding. An acceptable Cronbach’s Coefficient alpha value of 0.78 was received.

The dependent variable in this study was neonatal mortality, characterized as a binary variable expressing the outcome of live birth or neonatal death. Independent variables were categorized into three sections: I) Socio-demographic characteristics, including the mother’s age, education, marital status, family income, and sex of the newborn. II) Maternal characteristics including the number of ANC visits during pregnancy, gestational age at the first ANC visit, mother tetanus-toxoid vaccination, previous history of pregnancy complications, mothers taking supplements during pregnancy, mothers who take care of BMI, Mother’s BMI before pregnancy, history of UTI or PID, previous history abortion, mode of delivery, history complications during delivery, birth order, and birth intervals. III) Neonatal characteristics including the age of the newborn at discharge, gestational age of the baby, birth weight of the newborn, Apgar score at first five minutes, need for resuscitation, congenital abnormality, infectious diseases during hospitalization, taking BCG vaccine, initiated early breast-feeding, and final diagnose at discharge.

### Operational definitions

*Neonatal mortality* is defined as the passing away of a live-born newborn within the first 28 days of life.

*Congenital abnormalities* are structural or functional malformations that develop during fetal life. These illnesses, sometimes known as birth abnormalities, congenital disorders, or congenital deformities, happen during pregnancy and can be detected before, during, or after delivery [42].

*An APGAR score* is a rapid tool for clinicians to assess the health of all infants at 1 and 5 min after delivery and in response to resuscitation [43].

*Pelvic inflammatory disease (PID)* is a common cause of morbidity in young women and is generally caused by a sexually transmitted infection [44].

*Abortion* is the cessation of a pregnancy by removing or expulsing an embryo or fetus [45].

### Data collection procedure

A team of five BSc nursing professionals collected data at five selected hospitals following a comprehensive three-day training program. Before data collection, participants provided informed consent. Neonatal characteristics were extracted from medical record charts. At the same time, socio-demographic and maternal factors were collected through face-to-face interviews with the mothers or legal guardians, each lasting approximately 10–15 min. The senior researcher reviewed the collected data daily to ensure data accuracy and internal consistency.

### Data analyzes process

All collected data were checked for completeness and internal consistency by cross-checking and then coded and double-entered into Excel spreadsheet software and exported into the SPSS version (Chicago, IL) for data cleaning and analysis. Descriptive statistics were used for categorical variables presented by frequency and percentages, and means, standard deviations, maximum, and minimum presented as continuous data. The chi-square test was used to compare factors between neonatal death and alive at a *p*-value 0.05, with cross tab column %. Logistic regression was used to determine factors associated with neonatal mortality. Variables that were statistically significant in the univariable analysis used OR with 95%CI at *p*-value < 0.20, as recommended by Bursac et al., for the reasoning to detect all the significant risk factors under controlling for adjusting all possible confounders [46], were considered candidates in the multivariable regression model. The backward stepwise method was used in multivariable analysis. The multivariable model included all statistically significant independent variables, while non-significant ones were deleted one by one, starting with the largest *p*-value (above 0.05) until only significant variables (less than *p*-value 0.05) remained in the final model. The final model’s findings were presented as adjusted odds ratios (AOR) with 95% CI. The variance inflation factor (VIF) was employed to test for multicollinearity among independent variables in multiple regressions. A VIF ≥ 10 strongly indicates multicollinearity; for multiple regression models, the estimated VIF for all variables ranged from 1.01 to 1.42, which confirmed the absence of multicollinearity among variables. A Hosmer–Lemeshow goodness-of-fit test was used to assess the assumptions of good predictable variables in multivariate regression at the *p*-value > 0.05 [47]. All results were presented in tables and text.

## Results

### Socio-demographic characteristics

A total of 513 newborns participated in this study. Among them, 136 (26.5%) died, while 377(73.5%) were live newborns. Most of the participants 51.7% were male newborns, and 66.7% were mothers aged 19–35 years, with a mean age of 27.27 years (SD = 6.42), a maximum age of 43 years, and a minimum age of 16 years. Most of the mothers or caregivers (40.9%) were illiterate, 85.4% were married mothers, and 38.8% had a family income between 201–450$ per month (Table 1).

**Table 1:** Comparison of socio-demographic characteristics between neonatal death and alive among newborns admitted at selected hospitals in Mogadishu, Somalia 2023, (*n* = 513)

Characteristics	Total n (%)	Neonatal mortality		*p*-value
		**Death n (%)**	**Alive n (%)**	
**Total**	**513 (100.0)**	**136 (26.5)**	**377 (73.5)**	
**Mother’s age** (Years)				
≤ 18	80 (15.6)	25 (18.4)	55 (14.6)	0.297
19–35	342 (66.7)	92 (67.6)	250 (66.3)	
> 35	91(17.7)	19 (14.0)	72 (19.1)	
**Mother’s education**				
Illiterate	210 (40.9)	69 (50.7)	141 (37.4)	0.013^*^
Primary	108 (21.1)	24 (17.7)	84 (22.3)	
Secondary	130 (25.3)	34 (25.0)	96 (25.4)	
University	65 (12.7)	9 (6.6)	56 (14.9)	
**Mother’s marital status**				
Married	438 (85.4)	111 (81.6)	327 (86.7)	0.328
Divorce	47 (9.1)	15 (11.0)	32 (8.5)	
Widowed	28 (5.5)	10 (7.4)	18 (4.8)	
**Family income monthly** (US $)				
≤ 200	174 (33.9)	54 (39.7)	120 (31.8)	0.147
201–450	199 (38.8)	44 (32.4)	155 (41.1)	
> 450	140 (27.3)	38 (27.9)	102 (27.1)	
**Sex newborn**				
Female	248 (48.3)	77 (56.6)	171 (45.4)	0.024^*^
Male	265 (51.7)	59 (43.4)	206 (54.6)	

*Abbreviations*: *US* United States, *$* Dollar, *SD* Standard deviation

^*^P-value refers to the comparison between death and alive using the chi-square test at a significant level α < 0.05

### Maternal characteristics

More than two-thirds of mothers (62.3%) had less than 4 visits to ANC during pregnancy, 67.4% of mothers received tetanus-toxoid vaccine, 38.4% received folic acid supplement, 69.4% of mothers who unaware of their BMI before pregnancy, 62.0% delivered babies in SVD mode, 60.2% had babies with birth order 3 or above, and 73.3% had less than 24 months of pregnancy birth intervals (Table 2).

**Table 2:** Comparison of maternal characteristics between neonatal death and alive among newborns admitted at selected hospitals in Mogadishu, Somalia 2023, (*n* = 513)

Characteristics	Total n (%)	Neonatal mortality		*p*-value
		**Death n (%)**	**Alive n (%)**	
**No. of ANC visits during pregnancy**				
No visits	106 (20.7)	50 (36.8)	56 (14.9)	< 0.001^*^
< 4	320 (62.3)	72 (52.9)	248 (65.8)	
≥ 4	87 (17.0)	14 (10.3)	73 (19.3)	
**Gestational age at First ANC visit** (Weeks)				
≤ 12	205 (40.0)	72 (52.9)	133 (35.3)	0.002^*^
13–26	218 (42.5)	45 (33.1)	173 (45.9)	
> 26	90 (17.5)	19 (14.0)	71 (18.8)	
**Taking tetanus-toxoid vaccination of the mother**				
Yes	346 (67.4)	64 (47.1)	282 (74.8)	< 0.001^*^
No	167 (32.6)	72 (52.9)	95 (25.2)	
**Previous history of complications during pregnancy**				
Gestational Diabetic	33 (6.4)	5 (3.7)	28 (7.4)	0.016^*^
Hypertension	27 (5.3)	7 (5.1)	20 (5.3)	
Bleeding	23 (4.5)	11 (8.1)	12 (3.2)	
Anemia	65 (12.7)	20 (14.7)	45 (11.9)	
Others^**a**^	21 (4.0)	10 (7.4)	11 (2.9)	
None	344 (67.1)	83 (61.0)	261 (69.3)	
**Mothers taking supplement treatment during pregnancy**				
Folic acid tab only	197 (38.4)	35 (25.7)	162 (43.0)	< 0.001^*^
Ferro tab only	52 (10.1)	13 (9.6)	39 (10.3)	
Multivitamin tab only	35 (6.8)	7 (5.1)	28 (7.4)	
All Supplements	63 (12.3)	13 (9.6)	50 (13.3)	
None	166 (32.4)	68 (50.0)	98 (26.0)	
**Mothers taking care of their BMI before pregnancy**				
Yes	157 (30.6)	36 (26.5)	121 (32.1)	0.222
No	356 (69.4)	100 (73.5)	256 (67.9)	
**If yes, the BMI of the mother** (Kg/m^2^)				
Underweight (< 18)	19 (3.7)	6 (16.7)	13 (10.7)	0.604
Normal weight (18–24)	84 (16.4)	19 (52.8)	65 (53.7)	
Overweight & obesity (≥ 25)	54 (10.5)	11 (30.5)	43 (35.6)	
**History of UTI or PID during pregnancy**				
Yes	253 (49.3)	72 (52.9)	181 (48.0)	0.324
No	260 (50.7)	64 (47.1)	196 (52.0)	
**Previous history of abortion**				
Yes	168 (32.7)	48 (35.3)	120 (31.8)	0.461
No	345 (67.3)	88 (64.7)	257 (68.2)	
**Mode of delivery**				
SVD	318 (62.0)	90 (66.2)	228 (60.5)	< 0.001^*^
Instrumental assistant	53 (10.3)	23 (16.9)	30 (8.0)	
Cesarean Section(C-section)	142 (27.7)	23 (16.9)	119 (31.5)	
**History of complications during delivery**				
Prolong labor	151 (29.4)	38 (27.9)	113 (30.0)	0.108
Fetal distress	78 (15.2)	26 (19.1)	52 (13.8)	
Excessive bleeding	29 (5.7)	12 (8.8)	17 (4.5)	
Malposition	14 (2.7)	5 (3.7)	9 (2.4)	
None	241 (47.0)	55 (40.5)	186 (49.3)	
**Birth order of the child** (Number)				
1st order	102 (19.9)	31 (22.8)	71 (18.8)	0.270
2nd orders	102 (19.9)	31 (22.8)	71 (18.8)	
3rd orders and above	309 (60.2)	74 (54.4)	235 (62.4)	
**Birth interval pregnancy** (Months)				
< 24	376 (73.3)	101 (74.3)	275 (72.9)	0.765
≥ 24	137 (26.7)	35 (25.7)	102 (27.1)	

*Abbreviations*: *ANC* antenatal care, *BMI* body mass index, *UTI* urinary tract infection, *PID* pelvic inflammatory disease, *SVD* spontaneous vaginal delivery

^*^P-value refers to the comparison between death and alive using the chi-square test at a significant level α < 0.05

Others^a^ include (Premature rupture membrane, placenta previa, placenta abruption, and pre-eclampsia)

### Neonatal characteristics

Most newborns were ≤ 7 days old at discharge (74.5%), with a mean age of 6.2 days, SD = 5.03; the maximum age of newborns was 28 days, while the minimum age was 1 day. More than half (61.0%) were full-term between 37 to 41 weeks of gestational age, with a mean average of 36.5 weeks (SD = 4.2). 52.6% had normal birth weight between 2500–3999 g, with a mean average weight of 2569.9 g (SD = 938.46)0.36.5% had Apgar scores between 4 to 6. 70.0% those who needed resuscitation after delivery, 88.3% those who did not take BCG vaccine after delivery, and 78.4% those who did not take early breastfeeding after delivery (Table 3).

**Table 3:** Comparison of neonatal characteristics between neonatal death and alive among newborns admitted at selected hospitals in Mogadishu, Somalia 2023, (*n* = 513)

Characteristics	Total n (%)	Neonatal mortality		*p*-value
		**Death n (%)**	**Alive n (%)**	
**Age newborn at discharge** (days)				
≤ 7	382 (74.5)	106 (77.9)	276 (73.2)	0.278
8–28	131 (25.5)	30 (22.1)	101 (26.8)	
**Gestational age of the baby** (weeks)				
< 37	175 (34.1)	73 (53.7)	102 (27.1)	< 0.001^*^
37–41	313 (61.0)	51 (37.5)	262 (69.5)	
> 41	25 (4.9)	12 (8.8)	13 (3.4)	
**Birth weight newborn** (grams)				
Less than 2500	209 (40.8)	88 (64.7)	121 (32.1)	< 0.001^*^
2500–3999	270 (52.6)	42 (30.9)	228 (60.5)	
≥ 4000	34 (6.6)	6 (4.4)	28 (7.4)	
**Apgar scores first (5 Minutes)**				
≤ 3	186 (36.2)	50 (36.8)	136 (36.0)	< 0.001^*^
4–6	187 (36.5)	74 (54.4)	113 (30.0)	
≥ 7	140 (27.3)	12 (8.8)	128 (34.0)	
**Need resuscitation after delivery**				
Yes	359 (70.0)	114 (83.8)	245 (65.0)	< 0.001^*^
No	154 (30.0)	22 (16.2)	132 (35.0)	
**Congenital abnormality**				
Spina-bifida	14 (2.7)	5 (3.7)	9 (2.4)	0.759^**a**^
Hydrocephalus	17 (3.3)	6 (4.4)	11 (2.9)	
Congenital heart disease	21 (4.1)	6 (4.4)	15 (4.0)	
Others^**b**^	18 (3.5)	4 (2.9)	14 (3.7)	
None	443 (86.4)	115 (84.6)	328 (87.0)	
**Newborns having infectious diseases during hospitalization**				
Neonatal sepsis	121 (23.6)	36 (26.5)	85 (22.6)	0.001^*^
Neonatal tetanus	15 (2.9)	9 (6.6)	6 (1.6)	
Pneumonia	33 (6.4)	14 (10.3)	19 (5.0)	
Malaria	0 (0)	0 (0)	0 (0)	
Diarrheal	0 (0)	0 (0)	0 (0)	
None	344 (67.1)	77 (56.6)	267 (70.8)	
**Babies taking BCG vaccine after delivery**				
Yes	60 (11.7)	4 (2.9)	56 (14.9)	< 0.001^*^
No	453 (88.3)	132 (97.1)	321 (85.1)	
**Initiated early breastfeeding after delivery**				
Yes	111 (21.6)	14 (10.3)	97 (25.7)	< 0.001^*^
No	402 (78.4)	122 (89.7)	280 (74.3)	

*Abbreviation*: *BCG* bacille calmette-guerin

^*^P-value refers to the comparison between death and alive using the chi-square test at a significant level α < 0.05

^a^fisher’s exact test

Others^b^ include (Hirschsprung disease, anal atresia, cleft lip palate, down syndrome, hydronephrosis, hemoperitoneum, club foot, bladder exstrophy, gastroschisis, anal imperforate, and unilateral agenesis (right kidney)

### Final diagnosis at the discharge of newborns

A study found that birth asphyxia was the most common final diagnosis for newborns at discharge or death, accounting for 29% of cases. Neonatal sepsis was the second most common diagnosis at 16%, followed by premature birth, and RDS combined accounted for 10.3%. The highest neonatal death was among babies diagnosed with birth asphyxia at 21.3%, followed by premature birth at 13.2% and neonatal sepsis at 8.8% (Table 4).

**Table 4:** Final diagnosis of newborns at discharge or death, (*n* = 513) 2023

Characteristics	Total n (%)	Neonatal mortality	
		**Death n (%)**	**Alive n (%)**
**Final diagnoses of the newborn at discharge or death**			
Birth asphyxia	149 (29.0)	29 (21.3)	120 (31.8)
Congenital malformation	15 (2.9)	3 (2.2)	12 (3.2)
Hypoglycemia	9 (1.8)	0 (0)	9 (2.4)
Neonatal anemia	5 (1.0)	0 (0)	5 (1.3)
Neonatal jaundice	25 (4.9)	0 (0)	25 (6.6)
Neonatal sepsis	82 (16.0)	12 (8.8)	70 (18.6)
Neonatal tetanus	15 (2.9)	9 (6.6)	6 (1.6)
Pneumonia	23 (4.5)	5 (3.7)	18 (4.8)
Premature	42 (8.2)	18 (13.2)	24 (6.4)
Premature and pneumonia	9 (1.8)	9 (6.6)	0 (0)
Premature and RDS	53 (10.3)	24 (17.6)	29 (7.7)
Premature and sepsis	31 (6.0)	21 (15.4)	10 (2.7)
RDS	55 (10.7)	6 (4.4)	49 (13.0)

Some newborns had two or more diagnoses at the final discharge or death

*Abbreviation*: *RDS* Respiratory distress syndrome

### Prevalence of neonatal mortality and factors associated with neonatal mortality

In this study, the prevalence of neonatal mortality at selected hospitals in Mogadishu, Somalia, was found to be 26.5% [95%CI = 22.6–30.2]. While 513 neonates were admitted to the NICU of selected hospitals, three hundred seventy-seven neonates (73.5%) were discharged alive (Table 1).

In the univariable model, 17 variables whose values were below 0.20 in the crude odds ratio were entered into the multivariable logistic regression model to observe those that were significantly associated with neonatal mortality, including (mothers’ education, sex of the newborn, no. of ANC visits during pregnancy, gestational age at the first ANC visit, taking tetanus-toxoid vaccination of the mother, previous history of complications during pregnancy, mothers taking supplement treatment during pregnancy, mode of delivery, history of complications during delivery, birth order of the child, gestational age of the baby, birth weight newborn, Apgar scores first (5 Minutes), need resuscitation after delivery, newborns having infectious diseases during hospitalization, babies taking BCG vaccine after delivery, and initiated early breastfeeding after delivery (Table 5).

**Table 5:** Factors associated with neonatal mortality in univariable and multivariable analysis among newborns admitted at selected hospitals in Mogadishu, Somalia 2023, (*n* = 513)

Characteristics	OR (95%CI)	*p*-value	AOR (95%CI)	*p*-value
**Mother’s age** (Years)				
≤ 18	1.24 (0.73–2.09)	0.435		
19–35	1.00			
> 35	0.72 (0.41–1.25)	0.244		
**Mother’s education**				
Illiterate	3.05 (1.42–6.51)	0.004^*^		
Primary	1.78 (0.77–4.11)	0.178		
Secondary	2.20 (0.99–4.93)	0.054		
University	1.00			
**Mother’s marital status**				
Married	1.00			
Divorce	1.38 (0.72–2.65)	0.330		
Widowed	1.64 (0.73–3.65)	0.229		
**Family income monthly** (US$)				
≤ 200	1.21 (0.74–1.98)	0.452		
201–450	0.76 (0.46.1.26)	0.287		
> 450	1.00			
**Sex newborn**				
Female	1.57 (1.06–2.33)	0.025^*^	1.98 (1.22–3.19)	0.006^*^
Male	1.00		1.00	
**No. of ANC visits during pregnancy**				
No visits	4.66 (2.34–9.26)	< 0.001^*^	2.59 (1.05–6.45)	0.040^*^
< 4	1.51 (0.81–2.84)	0.196	1.39 (0.67–2.88)	0.371
≥ 4	1.00		1.00	
**Gestational age at First ANC visit** (Weeks)				
≤ 12	0.48 (0.31–0.74)	0.001^*^		
13–26	0.49 (0.28–0.89)	0.018^*^		
> 26	1.00			
**Taking tetanus-toxoid vaccination of the mother**				
Yes	1.00		1.00	
No	3.34 (2.22–5.03)	< 0.001^*^	1.82 (1.01–3.28)	0.045^*^
**Previous history of complications during pregnancy**				
Gestational Diabetic	0.56 (0.21–1.50)	0.250		
Hypertension	1.10 (0.45–2.69)	0.834		
Bleeding	2.88 (1.23–6.78)	0.015^*^		
Anemia	1.39 (0.78–2.50)	0.259		
Others	2.86 (1.17–6.97)	0.021^*^		
None	1.00			
**Mothers taking supplement treatment during pregnancy**				
All Supplements	1.00			
Folic acid tab only	0.83 (0.41–1.69)	0.610		
Ferro tab only	1.28 (0.53–3.08)	0.578		
Multivitamin tab only	0.96 (0.34–2.69)	0.940		
None	2.67 (1.35–5.29)	0.005^*^		
**Mothers’ taking care of their BMI before pregnancy**				
Yes	1.00			
No	1.31 (0.85–2.04)	0.223		
**If yes, the BMI of the mother** (Kg/m^2^)				
Underweight (< 18)	1.58 (0.53–4.72)	0.413		
Normal weight (18–24)	1.00			
Overweight & obesity (> 25)	0.88 (0.38–2.02)	0.755		
**History of UTI infection or PID during pregnancy**				
Yes	1.22 (0.82–1.80)	0.324		
No	1.00			
**Previous history of abortion**				
Yes	1.17 (0.77–1.77)	0.461		
No	1.00			
**Mode of delivery**				
SVD	1.00		1.00	
Instrumental assistant	1.94 (1.07–3.52)	0.029^*^	3.01 (1.38–6.56)	0.006^*^
Cesarean Section(C-section)	0.49 (0.29–0.81)	0.006^*^	0.50 (0.27–0.93)	0.027^*^
**History of complications during delivery**				
Prolong labor	1.14 (0.71–1.83)	0.596		
Fetal distress	1.69 (0.97–2.96)	0.065		
Excessive bleeding	2.39 (1.08–5.30)	0.033^*^		
Malposition	1.88 (0.61–5.84)	0.276		
None	1.00			
**Birth order of the child** (Number)				
1st order	1.00			
2nd orders	1.00 (0.55–1.82)	1.000		
3rd orders and above	0.72 (0.44–1.19)	0.197		
**Birth interval pregnancy** (months)				
< 24	1.07 (0.69–1.67)	0.765		
≥ 24	1.00			
**Age newborn at discharge** (days)				
≤ 7	1.29 (0.81–2.06)	0.279		
8–28	1.00			
**Gestational age of the baby** (weeks)				
< 37	3.68 (2.41–5.62)	< 0.001^*^	1.99 (1.00–3.94)	0.049^*^
37–41	1.00		1.00	
> 41	4.74 (2.05–10.98)	< 0.001^*^	4.82 (1.64–14.16)	0.004^*^
**Birth weight newborn** (grams)				
Less than 2500	3.95 (2.57–6.06)	< 0.001^*^	4.82 (2.34–9.95)	< 0.001^*^
2500–3999	1.00		1.00	
≥ 4000	1.16 (0.45–2.98)	0.753	0.85 (0.27–2.72)	0.786
**Apgar scores first (5 Minutes)**				
≤ 3	1.78 (1.15–2.76)	0.010^*^		
4–6	0.26 (0.13–0.50)	< 0.001^*^		
≥ 7	1.00			
**Need resuscitation after delivery**				
Yes	2.79 (1.69–4.62)	< 0.001^*^	2.78 (1.51–5.13)	0.001^*^
No	1.00		1.00	
**Congenital abnormality**				
Spina-bifida	1.59 (0.52–4.83)	0.418		
Hydrocephalus	1.56 (0.56–4.30)	0.394		
Congenital heart disease	1.14 (0.43–3.01)	0.790		
Others	0.82 (0.26–2.53)	0.723		
None	1.00			
**Newborns having infectious diseases during hospitalization**				
Neonatal sepsis	1.47 (0.92–2.34)	0.105	2.24 (1.26–3.98)	0.006^*^
Neonatal tetanus	5.20 (1.79–15.07)	0.002^*^	16.03 (3.69–69.49)	< 0.001^*^
Pneumonia	2.56 (1.23–5.33)	0.012^*^	4.06 (1.60–10.31)	0.003^*^
None	1.00		1.00	
**Babys taking BCG vaccine after delivery**				
Yes	1.00			
No	5.76 (2.05–16.19)	0.001^*^		
**Initiated early breastfeeding after delivery**				
Yes	1.00		1.00	
No	3.02 (1.66–5.49)	< 0.001^*^	2.28 (1.12–4.66)	0.023^*^

1 = Reference

^*^Significant level at α = 0.05

In multivariable analysis, nine variables were found to be associated with neonatal mortality. Female newborns had a greater chance of neonatal mortality than males, with AOR = 1.98 times (95%CI = 1.22–3.19). Neonates with their mothers who did not attend ANC visits had a greater risk of neonatal mortality than those who attended it, with AOR = 2.59 times (95%CI = 1.05–6.45). Neonates with their mothers who did not take tetanus toxoid vaccination had greater odds of neonatal mortality than those who took it, with AOR = 1.82 times (95%CI = 1.01–3.28). Neonates with mothers who delivered in the instrumental assistant mode (95%CI = 1.38–6.56) were 3.01 times more likely to neonatal mortality than those who delivered in the SVD mode. In contrast, those who delivered in C-section (95%CI = 0.27–0.93) were 0.50 times less likely to neonatal mortality than those who delivered in SVD mode. Neonates who had neonatal sepsis, tetanus, and pneumonia diseases during hospitalization had a greater chance of neonatal mortality than those who did not have infectious diseases, with AOR = 2.24 times (95%CI = 1.26–3.98), AOR = 16.03 times (95%CI = 3.69–69.49), AOR = 4.06 times (95%CI = 1.60–10.31), respectively (Table 5).

Premature (95%CI = 1.00–3.94) and postmature (95%CI = 1.64–14.16) neonates, 1.99 and 4.82 times, respectively, were more likely to neonatal mortality than full-term. Neonates with a birth weight of less than 2500 g had a greater chance of neonatal mortality than those born weighing 2500 to 3999 g, with AOR = 4.82 times (95%CI = 2.34–9.95). Neonates who needed resuscitation had a greater chance of neonatal mortality than those who did not, with AOR = 2.78 times (95%CI = 1.51–5.13). Neonates who did not initiate early breastfeeding after delivery had a greater chance of neonatal mortality than those who initiated early breastfeeding, with AOR = 2.28 times (95%CI = 1.12–4.66) (Table 5).

## Discussion

Neonatal mortality is a significant public health concern, particularly in low and middle-income countries. Mogadishu, Somalia, currently grapples with a high burden of neonatal mortality. The prevalence of this study was found to be high and relatively comparable to a previous study conducted in India [19]. However, it is higher than studies conducted in Asmara, Eritrea [26], Cameroon [30], Southern and Northern Ethiopia [27, 48], Eastern Ethiopia [49], Congo [50], and Somali Regions of Ethiopia [32, 51]. On the other hand, this prevalence is lower than the studies conducted in Southwest Ethiopia [21] and Ghana [52]. This variation in prevalence may be attributed to various factors, including cultural beliefs, maternal knowledge, attitude, and practice of prenatal and postnatal care, socio-economic conditions, access to healthcare, availability of medical equipment and supplies, environmental conditions, and population. The findings of this study could be helpful for healthcare providers, policymakers, and NGOs in improving healthcare infrastructure, ensuring adequate prenatal care, and implementing educational campaigns for mothers and healthcare workers.

According to the study, the final diagnosis revealed that 21.3% of newborns died due to birth asphyxia, 13.2% were premature, and 8.8% were neonatal sepsis. This finding is consistent with studies conducted in Cameroon [53] and the Somali region of Ethiopia [32], which also identified premature birth, neonatal sepsis, and birth asphyxia as the most common causes and risk factors of neonatal death. These conditions can be life-threatening if not managed promptly and effectively due to various complications that may arise. Furthermore, underdeveloped nations face a higher neonatal mortality rate due to inadequate focus on pregnancy, intrapartum, and early newborn medical care practices. The insufficient efforts to reduce neonatal mortality in these regions have contributed to the high death rates [54].

Based on the study’s final model, it was discovered that neonatal mortality is significantly related to 9 variables. Interestingly, the study found that female newborns are more likely to experience neonatal mortality compared to male newborns. This finding is supported by a study conducted in Urban Pakistan, which revealed that female neonates are at a higher risk of neonatal mortality than males, particularly during the late neonatal period (8–28 days) [22]. Furthermore, the study highlights that female newborns tend to have slightly higher birth asphyxia and birth weight of ≥ 4000gr compared to their male counterparts. However, the study also discovered that mothers with female newborns attend ANC visits less than four times due to cultural bias. This may be attributed to a preference for males as firstborns. A similar study in China revealed that 93% of nulliparous women prefer males as their first baby due to cultural and social influences [55]. This finding is inconsistent with other studies conducted in Ghana [52], Nepal [56], India and Pakistan [57], and China [58], which reported that male neonates have a higher risk of neonatal mortality due to biological phenomena. It is worth noting that the risk difference between genders may vary depending on the population being studied.

The findings of this study highlight a concerning trend: newborns born to mothers who did not receive the tetanus toxoid vaccine face a significantly higher risk of neonatal mortality compared to those whose mothers received the vaccine. This is corroborated by a similar study conducted in India, which found that 20% of neonatal deaths could be attributed to mothers not receiving at least a single dose of the tetanus toxoid vaccine during pregnancy [59]. Furthermore, it is essential to note that Sub-Saharan Africa has the lowest tetanus toxoid vaccine coverage globally [60]. In the study area, it becomes evident that the coverage of maternal tetanus vaccination is inadequate due to a lack of awareness and prevailing cultural beliefs. This deficiency in vaccine coverage elevates the risk of neonatal tetanus. The absence of a single vaccine dose in mothers can potentially lead to the transmission of tetanus during childbirth, resulting in life-threatening complications, including neonatal tetanus, as supported by similar previous studies in Ghana [61] and India [59].

A notable finding from this study was that newborns diagnosed with sepsis, tetanus, and pneumonia during hospitalization are at a greater risk of neonatal mortality than those without any infections. According to research conducted in Zambia, sepsis is responsible for 43% of newborn deaths [62]. Due to an undeveloped immune system and small airways, sepsis and pneumonia can lead to severe organ damage and respiratory failure in newborns, making it a potential adverse outcome. Neonatal tetanus is also a high mortality risk due to underdeveloped immune systems and muscle spasms, which can result in respiratory failure and death. A study in Uganda reported that neonatal tetanus cases caused high mortality rates among newborns [63], supporting this observation. Furthermore, poor people in the study area often use unsterilized tools or contaminated substances to cut the umbilical cord, increasing the risk of bacteria entering the baby’s body and potentially leading to adverse outcomes, including death [64]. Consequently, it is necessary to enhance hygienic practices, disease prevention and treatment guidelines, and the training of healthcare workers to reduce the risk of neonatal death during hospitalization. Substantially, the country requires programs to prevent and control infections due to the high prevalence of infectious diseases and the risk of outbreaks, as demonstrated in previous studies [65–68]. This could play a pivotal role in reducing the number of deaths among newborns. This study’s findings are in line with those conducted in Pakistan [22], Botswana [62], and Ethiopia [54, 69].

This study unveiled that both premature and postmature newborns faced a heightened risk of neonatal mortality compared to their full-term counterparts. A study conducted in Northern Ethiopia found that premature infants were at a greater risk of mortality when contrasted with full-term infants [70]. This heightened vulnerability in premature babies can be attributed to their underdeveloped immunity and increased susceptibility to infections, which, in turn, may result in life-threatening complications such as respiratory distress, brain hemorrhage, and infections. Conversely, postmature infants, owing to their larger size and reduced amniotic fluid levels, may encounter health challenges like meconium aspiration, hypoglycemia, and jaundice. A prior study in Ghana also underscored the significance of gestational age as a risk factor for neonatal mortality [71]. Enhancing standardized guidelines for the care of premature babies and ensuring the accessibility of adequate surfactant treatment could substantially diminish the risk of premature mortality, as corroborated by analogous studies conducted in Ethiopia [54, 69], Ghana [71], and India [72]. Furthermore, our study identified that neonates with a birth weight below 2500 g faced a greater risk of neonatal mortality compared to those with normal birth weights. A preceding study in Ethiopia similarly observed that low birth weight in small-sized neonates heightens their vulnerability to illnesses due to their limited immune response [28]. This susceptibility can be attributed to their underdeveloped organ systems during the initial weeks of life, making them more prone to infections and complications [73], which is consistent with findings from previous studies in Ethiopia [28, 40, 74] and Nepal [73].

In this study, it was observed that neonates born to mothers who did not attend antenatal care (ANC) visits faced a higher risk of neonatal mortality compared to those whose mothers did attend ANC visits. This result aligns with several studies conducted in various regions of Ethiopia [27, 32, 54]. The plausible explanation behind this is that mothers who miss ANC visits might overlook critical health interventions, which might increase the risk of complications and even death for their babies. Hence, regular ANC visits are crucial for the timely detection and management of complications, promoting maternal health, preparing for childbirth, and providing necessary health interventions and education.

This study found that neonates born to mothers who underwent instrumental deliveries were at a higher risk of neonatal mortality in comparison to those born via spontaneous vaginal delivery (SVD). A study in Ethiopia discovered that 45.7% of neonates delivered through instrumental methods experienced complications and had low APGAR scores, which are known risk factors for neonatal mortality [75]. The use of instruments during delivery, such as forceps or vacuum extraction, was associated with an increased risk of neonatal mortality. Furthermore, a prior study in the same area reported that nearly half of pregnant women had undergone female genital mutilation (FGM) [36]. This practice can lead to scarring and narrowing of the vaginal opening, necessitating instrumental deliveries. This, in turn, may elevate the risk of adverse outcomes, including mortality. However, when performed by experienced healthcare professionals, the associated risk can be minimized. In contrast, cesarean-section (C-section) delivery may serve as a protective factor against neonatal mortality in specific situations, mainly when complications arise during vaginal delivery. It may also be recommended for mothers with conditions such as HIV, herpes, or placenta previa to mitigate the risk of transmitting infections to the newborn or prevent hemorrhage and potential mortality. Research conducted in Ethiopia indicated that mothers who delivered via C-section had a 66% lower risk of newborn mortality compared to those who underwent SVD, primarily due to prolonged labor and other complications associated with SVD [27]. These findings were substantiated by studies conducted in Western Oromia, Ethiopia [75], Canada [76], and Southern Ethiopia [27].

Neonates who required resuscitation had a higher risk of neonatal mortality compared to those who did not need resuscitation. This finding aligns with a study conducted in Southern Ethiopia [27]. The reason behind this could be that if the newborn is not resuscitated quickly and efficiently when they are not breathing adequately or have a low heart rate, it can lead to hypoxia and permanent brain damage. Moreover, early successful resuscitation in the delivery room can help minimize birth-related mortality [77, 78]. In Tanzania, a study revealed that delayed resuscitation significantly impacted mortality rates, with 69% of the neonates who required CPR dying due to delayed interventions [79]. Nonetheless, in the study area, the risks can be reduced by providing early resuscitation and improving health facilities, which can be achieved through maternal and child health centers (MCHs), clinic centers, and birth attendants. Moreover, this finding showed that neonates who did not initiate early breastfeeding after delivery had greater odds of neonatal mortality than those who initiated early breastfeeding. This finding is consistent with studies conducted in Ethiopia [27], Indonesia [80], and India [81]. The rationale behind this correlation is that delaying the onset of early breastfeeding can result in severe complications, ultimately leading to neonatal mortality. However, within the scope of the current study, it appears that insufficient awareness of antenatal care follow-up and healthcare resources may contribute to mothers postponing colostrum feeding for their infants. Furthermore, to mitigate neonatal mortality, it is imperative to initiate breastfeeding promptly [82].

## Conclusion

The study highlights that neonatal mortality is a major issue in Mogadishu, Somalia, with a high prevalence of mortality among female newborns, mothers who did not attend ANC visits during pregnancy, and those who did not receive tetanus toxoid vaccination. The study also identified several risk factors that significantly contribute to neonatal mortality, including assisted instrumental delivery mode, newborns who had sepsis, tetanus, and pneumonia, those born prematurely or postmature, those with birth weights less than 2500gr, newborns who needed resuscitation after delivery, and those who did not initiate early breastfeeding after delivery. Interestingly, mothers who delivered through C-section mode were found to be a protective factor of neonatal mortality. The study further revealed that birth asphyxia, premature birth, and neonatal sepsis were the leading causes of newborn deaths, accounting for over forty percent of the deaths. To reduce neonatal mortality rates, it is recommended to provide training to health personnel in programs like Neonatal Resuscitation Program (NRP) and Helping Babies Breathe (HBB) for neonatal resuscitation. Furthermore, developing a national protocol for neonatal sepsis and promoting antenatal visits through health educational programs using local media like TV and radio could play a pivotal role in reducing the number of deaths at a national level. Therefore, it is crucial to enhance antenatal and postnatal care, provide emergency obstetric and neonatal services, and implement up-to-date practices like early breastfeeding, infection control and prevention, and maternal education to improve neonatal outcomes and reduce mortality. The study calls for further research to understand risk factors contributing to neonatal mortality, such as premature birth, birth asphyxia, and neonatal sepsis. Moreover, it also suggests evaluating Mogadishu’s healthcare systems’ capacity to address neonatal health needs, identifying gaps in infrastructure, trained professionals, medical supplies, and access to prenatal and neonatal care services.

### Study strength and limitation

One of the study’s strengths is that it was conducted in multiple centers, including both public and private hospitals, that serve as referral hospitals. Moreover, to the best of our knowledge, it is the first study of its kind conducted in Mogadishu, Somalia, which utilized primary data. However, the study’s limitation is that it used a cross-sectional study design. As a result of the design’s nature, it was impossible to determine the causal relationship between the factors studied and their impact on neonatal mortality.

## Data Availability

The data set used and analyzed in the current work are available from the corresponding author upon reasonable request.

## Abbreviations

NM: Neonatal Mortality
SDG: Sustainable Development Goals
WHO: World Health Organization
NICU: Neonatal Intensive Care Unit
PID: Pelvic Inflammatory Disease
UTI: Urinary Tract Infection
BCG: Bacille Calmette-Guerin
FGM: Female Genital Mutilation
CPR: Cardiopulmonary Resuscitation
SD: Standard Deviation
SVD: Spontaneous Vaginal Delivery.
RDS: Respiratory Distress Syndrome
NRP: Neonatal Resuscitation Program
HBB: Helping Babies Breathe
